# Biosensing and Biosensors—Terminologies, Technologies, Theories and Ethics

**DOI:** 10.1111/gec3.70007

**Published:** 2024-11-18

**Authors:** Jan Misera, Johannes Melchert, Tabea Bork‐Hüffer

**Affiliations:** ^1^ Department of Geography University of Innsbruck Innsbruck Austria

**Keywords:** biosensing, biosociality, critical neurogeographies, ethics of care, eye‐tracking, mobilities paradigm, more‐than‐representational theories, wearable biosensors

## Abstract

Which biosensing technologies are geographers using in their research, and what exactly do they measure? What are the theoretical origins of geographic interests in biosensing? This article provides an overview of the variety of biosensors applied in biosensing research, tracks the theoretical debates and roots of geographic engagement with biosensing, and discusses the potentials, limitations and ethical implications of applying biosensors. We critically reflect on the varied terminologies that have been used to describe a rapidly evolving array of biosensing technologies and methodologies and suggest a common understanding for key terms such as “biosensing” (technologies or methodologies), “biosensors,” “wearable biosensors” and “biosignals.” We offer an overview of the broader theoretical debates that have inspired geographers turn to biosensing, including behavioral geography, more‐than‐representational theory, critical neurogeography, the mobilities and biosociality paradigms, and visual geographies. These have called for methodologies that can capture affects neglected in representational research, follow people, things and technologies as they are mobile in space and time, investigate the links between brain, cognition and biopolitics or attend to visualities in everyday life. Although geographers have so far engaged with a limited number of the ever‐growing variety of available (bio‐)sensors, the development and application of biosensing methodologies is vibrant, highly diverse and very promising for diverse geographical research questions and fields. Going forward, we particularly encourage experimentation with eye‐trackers, which come closest to measuring instantaneous responses to environmental stimuli and offer interesting opportunities for the analysis of social and material environments through the visual data they create. Finally, we conclude with a call for a stronger emphasis on data ethics, procedural ethics and ethics of care in biosensing, which have so far received too little attention in these often interdisciplinary and complex biosensing research endeavors.

## 1 Introduction

Despite the growing interest in non‐invasive biosensors in environmental psychology, neuroscience, and related fields, the scholarly community working with biosensing methodologies in geography and spatial sciences remains small. Varied terminologies have been used to describe a rapidly evolving array of biosensing technologies and methodologies.

Of most interest to geographers and related scholars are wearable biosensors integrated into devices such as smartwatches, wristbands, clothing, earbuds or eye‐trackers (dos Santos et al. 2021; Mück et al. 2019; Ray et al. 2019). They enable the recording, measurement, and monitoring of affective responses in different environments and foster the understanding of human (environmental) perception, social interactions, and affects (Pykett 2018). Over the past decade, wearable biosensors have become more affordable, less invasive and more suitable for everyday use. Nonetheless, efforts to measure affective‐emotional responses through (ever evolving) sensors have a long history, originating from disciplines such as medicine, clinical research, psychology, engineering, biology, chemistry, physics, computer sciences and consumer sciences (Lowe 2007; Naresh and Lee 2021). Accordingly, fields of application of biosensors are broad, ranging from environmental monitoring, healthcare monitoring, disease identification, food quality management to consumer attention (Paiva et al. 2023).

As Spinney (2015, 239) postulates, biosensing could enable the gathering of “data in a quantitative form that attempts to avoid the subjectivism of [previous behavioral geographical] approaches which seek to interpret bodily experience through verbal and visual recollections.” It allows the analysis of entanglements of bodies, practices, technologies, and materialities in situ and in real time (Hesse‐Biber and Johnson 2013; Kaufmann et al. 2021). Recent technological advancements of biosensors and the mobile devices in which they are integrated, along with new theoretical engagements, suggest that it may only be a matter of time until more and more geographers become interested in experimenting with different biosensors in various in situ settings and fields of study.

Geographic interest in biosensing has been inspired by several broader theoretical debates. When envisioning future interdisciplinary engagements between geographic reasoning and biosensing, it is important to acknowledge that previous theoretical and methodological endeavors with neuroscience and behavioral science have faced widespread criticism and intra‐disciplinary “marginalization” (Bunnell, Ng, and Yeo 2023, 2) since the 1980s. It is therefore to no surprise that many geographers have long distanced themselves from a deeper engagement with perspectives from the neurosciences and behavioral sciences, despite shared interests and areas of inquiry (Bunnell, Ng, and Yeo 2023). However, this has not prevented all scholars from venturing into what Pykett (2018, 154–155) refers to as “mind/brain/world interactions.” From feminist and poststructuralist perspectives on the perception of spatial environments to non‐/or more‐than‐representational theory, affect theory and more recent attempts to critically approach a new form of neurogeography, various engagements—despite perceived marginalization—have found and developed new pathways without reiterating some of the blind spots of behavioral geography.

This article pursues two main aims and thereby addresses two audiences. First, we offer suggestions for a—so far missing—standardized biosensing terminology, that differentiates specific technologies and biosensors and debate their varied potentials and limitations. Second, we provide a concise overview of the different theoretical strands and studies in the field to offer a comprehensive overview of the historicities of theoretical debates that have inspired geographers to experiment with biosensing methodologies. Thus, this article speaks both to the biosensing community, to whom we present a discussion on the delimitation of the filed, and to readers new biosensing who seek an introduction of the terminologies, technologies, and theories.

To achieve these goals, we begin with an overview of the variety of technologies and (bio‐)sensors applied in biosensing research, debating their respective potentials and limitations and suggest a common terminology that may support interested scholars navigate this complex field (Section 2). We then trace the theoretical debates and roots of geographic engagement with biosensing, while providing examples of applications in the field (Section 3). This is followed by a discussion of the potentials but also limitations of specific biosensors (Section 4). To conclude and by providing an outlook we call for the careful and ethically‐reflected implementation of these emerging technologies (Section 5).

## 2 Terminologies and Technologies: Identifying a Common Approach Toward Delimiting (Wearable) Biosensing

We note a great diversity of terminologies being used in various disciplines and fields of application of biosensing, referring to technologies, biosensors as well as biosensing as methodology. The beginnings of biosensing, and thus its terminology do not originate in geography but have been adopted by geographers in similarly diverse ways. Consequently, there is a need to critically reflect on this terminology and to foster a common understanding. Therefore, this section aims to discuss the following key terms: “biosensing” (technologies or methodologies), “biosensors,” “wearable biosensors” and “biosignals.” What may at first glance appear to the reader as a list of terms that each have a unique understanding is, on closer inspection, a collection of umbrella terms with varying definitions and parallel uses across disciplines such as biology, chemistry, physics, engineering or medicine as well as geography (Kulkarni, Ayachit, and Aminabhavi 2022; Lowe 2007) within diverse fields of applications (e.g., affective responses to diverse environments, disease detection, toxin detection, food safety, drug delivery, pathogen delivery, environmental monitoring or healthcare monitoring (Kulkarni, Ayachit, and Aminabhavi 2022; Q. Lin et al. 2021)).

It is necessary to briefly introduce some of the basic functionalities common to most biosensing technologies, which can be used as a foundation for their delimitation. We propose the following approach to differentiate biosensing technologies according to:

- the “biosensors”/“sensors” used; and
- the components of the (bio‐)sensor itself.

Biosensors have been developed to detect specific biological, physical and chemical reactions, which they convert into measurable signals (Bollella and Katz 2020; Khan and Song 2020). Most biosensors are nowadays characterized by their selectivity, reproducibility, stability or greater independence of environmental factors (e.g., pH, temperature), sensitivity, and reusability (Bhalla et al. 2016; Lowe 2007). Common biosensing technologies include electrochemical biosensors (ECG, EEG, EMG, EDA/GSR, pH‐sensors, glucose‐sensor), optical biosensors (eye‐tracking, pulse oximeters, photoplethysmography [PPG], near‐infrared thermometer) as well as mechanical biosensors (accelerometer, gyroscope, magnetometer). Typically, biosensors consist of most of the following components:

- “Analyte”—the substance of interest or target analyte that needs to be detected (e.g., glucose, alcohol, lactose).
- “Bioreceptors”—specific molecules that recognize the analyte by generating a signal (e.g., light, heat, pH). Common bioreceptors are enzymes, antibodies, cells or DNA. The process is called biorecognition.
- “Transducer”—converts the detected signal or biorecognition event into another measurable signal (e.g., electrical, electrochemical, optical, thermal). It is therefore a “process of energy conversion (…) known as signalisation” (Bhalla et al. 2016, 1).
- “Electronics”—process and amplify the different signals (from analog to digital data) to display them on a screen.
- “Display”—projects the digital signal on a screen/display in a user‐friendly manner through numeric, graphic, tabular or imagery data output.

(Bhalla et al. 2016; Kim et al. 2019; Naresh and Lee 2021; Perumal and Hashim 2014).

Being applied by scholars from various disciplines, biosensors are nowadays developed for highly specific fields of application. Many of these specialized biosensors might therefore not be of relevance for research contexts of interest to (human) geographers and related scholars. Biosensors developed for in vitro or single‐use measurements, for example, are not necessarily designed to be wearable or tied to continuous monitoring which would be a requirement for out of the lab purposes. Of particular interest are non‐invasive wearable biosensors, which are integrated into small electronic devices and can be worn or attached to the body (Liu et al. 2018; Sharma et al. 2021). To date, wearable biosensors have been applied in life sciences, healthcare (monitoring, prevention, treatment), sport analytics, psychology and geography (Giannakakis et al. 2022; Kim et al. 2019; Pantelopoulos and Bourbakis 2010; Skaramagkas et al. 2021; Smith, Li, and Tse 2023; Ye et al. 2020). Since the introduction of smartphones in 2007 (Kim et al. 2019; Pantelopoulos and Bourbakis 2010), accompanied by improvements of microelectronics, in micro‐ and nano‐sensor manufacturing and miniaturization processes (Liu et al. 2018; Naresh and Lee 2021), wearable biosensors are increasingly suited for real‐time, out‐of‐lab measurements and monitoring. They can be differentiated according to:

- the stationary or mobile device into which (wearable) biosensors are integrated; and
- the field of application.

Wearable biosensors are increasingly compact, capable of real‐time measurement, minimally invasive and require little to no calibration. Some wearable biosensors can measure biochemical signals, commonly biofluids such as salvia, sweat, tears and provide insights on diverse conditions such as wellness and health status, aiding medical practitioners in managing chronic diseases (Ates et al. 2022; Kim et al. 2019; G. Li and Wen 2020; Liu et al. 2018). In geographical research, wearable biosensors are common, with all three types—electrochemical biosensors, optical biosensors, mechanical biosensors—being applied. Through the integration of several biosensors—such as electrodermal activity (EDA) sensors and photoplethysmography (PPG) sensors—the devices can measure multiple body responses at the same time. Photoplethysmography (PPG), an optical based near infrared technique, detects volumetric changes in blood circulation and is commonly integrated into devices such as smartwatches (Allen 2007; Giannakakis et al. 2022).

Body responses (physiological or somatic) which are commonly measured are skin conductance, respiration and breath rate, heart activity, blood pressure, electrodermal activity, electrical activity in the brain, electrical activity in the muscles and body posture/movements and eye movements. Those that can be measured or detected by (wearable) biosensors are generally being referred to as biosignals. They are described “as a description of a physiological phenomenon” (Kaniusas 2012, 1). Biosignals related to emotional arousal, attention and cognitive processes (cf., Osborne and Jones 2017; Pykett 2022) regulated by the autonomic nervous system (Eckstein et al. 2017; Kyriakou et al. 2019) are increasingly measurable through wearable biosensors integrated in small‐scale devices such as smartwatches or wristbands.

Less considered, but also attributable to the category of wearable biosensors, are optical (infrared) sensors integrated in (mobile) eye‐tracking devices (Sun et al. 2022). Like the previously mentioned biosignals, eye movements and pupil dilations are regulated via the sympathetic nervous system (SNS) and parasympathetic nervous system (PNS) as part of the autonomic nervous system (ANS). Their measures are being referred to as physiological and physical biosignals (Giannakakis et al. 2022). Hence, and in line with other scholars, we suggest categorizing them as biosensors too (Skaramagkas et al. 2021; Wu et al. 2022; Zheng et al. 2023). Novel mobile eye‐trackers, are capable of measuring eye movements in dynamic outdoor settings, could be of particular interest to geographers, as they provide “valuable information for one's higher cognitive function and state of affect” (Skaramagkas et al. 2021, 1).

As wearable biosensors are continually developed to improve out‐of‐the‐lab applications, and as research integrates them into their methodological designs, it might be fruitful for scholars to establish a common terminology, especially given the variety of terms used in parallel. In summary, (wearable) (bio‐)sensors are integrated into a variety of devices, such as smartphones, smartwatches, eye‐tracking glasses and stationary systems. We therefore suggest the following terminology:

- “Biosensing (technologies)” for the broader technology.
- “Biosensing methodologies” and “mobile biosensing methodologies” for mobile methodological applications in the field.
- “Biosensors” when referring to the sensors integrated into devices
- “Wearable biosensing” or “wearable biosensors” for devices designed for mobile/non‐stationary use while also considering their potential for stationary applications.
- “Biosignals” as physical, (bio)‐chemical changes providing essential information on human body processes.

## 3 Theories: Retracing the Theoretical and Epistemological Roots to Geographic Interest in Biosensing Methodologies

While the surge of the discipline's interest in biosensing might still be framed as a relatively recent development, its rich and diverse theoretical and epistemological roots are anything but new. This section, therefore, highlights the underlying meta‐theoretical perspectives that have influenced and initiated calls for new epistemologies and experimentation with biosensing methodologies in the first place. By tracing and (inter)‐connecting theorizations of and discussions on affects, mobilities, the visual and the brain, with biosensing practices we want to emphasize some of their epistemological commonalities and provide foundations for an emerging, yet still fragmented, field.

### 3.1 Measuring Affects: From Behavioral Geography to More‐Than‐Representational Theory

When searching for the very roots of geographic interest in what nowadays can be measured with biosensing it might be of use to begin with what became known as behavioral geography, a subdiscipline which thrived from the 1960s–1980s (cf., Argent 2017; Downs 1970; Gold 1980; Golledge and Stimson 1987). Through the analysis of patterns and variabilities and the creation of complex models and schemes, behavioral geographers sought to understand how certain spaces and environments influenced human perception of them and vice versa. Although evolving itself out of criticism on abstract and deterministic quantitative spatial sciences of the 1950s and 1960s, concerns were soon raised toward behavioralism, too. The concerns centered around behavioral geography's tendency toward behaviorism as well as mechanistic model‐thinking and its dichotomic understanding of human behavior and neglect of agency (Argent 2017). Prevalent amongst many criticisms was the notion that their explanations of real‐world occurrences were too often based upon problematic generalizations of small‐scale laboratory experiments (Cox 1981; Pile 1993; Pykett 2018).

However, despite being substantially challenged and mostly replaced by both practice theory and poststructuralist thinking and their focus on human practice, agency, discourse and power, and therewith representations, an interest lingered in those subconscious, momentary aspects that might not be communicated through words and emerge during the coming together of bodies and materialities in complex situational spaces and environments. Ever since N. Thrift's (1996) provocative critique of “traditional researchers” overemphasis on representations and the “mesmerized attention given to texts and images” (Waterton 2018, 91) a growing body of scholars has begun to reassess some of the discipline's previous engagements with neuroscientific explanations of human behavior and affect under the theoretical perspective of non‐representational theory (NRT). NRT encompasses a host of differing perspectives, theories, and methodologies with roots not only in human geography but also across the humanities, social sciences, arts and philosophy (Vannini 2015). In relating to theoretical strands such as poststructuralism, new materialism or postphenomenology, NRT can be described as a call to reimagine social and humanities sciences' approach to research and theory‐crafting as a more open‐minded, imaginative methodological practice—one that more strongly relates to the dynamic, diverse and non‐human character of the world, respects experimentation and is attentive to incompleteness and speculation (N. J. Thrift 2008; Williams 2020). NRT pays equal tribute to materialities, corporealities and socialities. By placing more emphasis on what H. Lorimer (2005, 83) referred to as “self‐evidently more‐than‐human, more‐than textual, multisensual worlds,” NRT underlines the importance of affects as pre‐cognitive, momentary subconscious elements in human perception that humans do not manage to verbalize or otherwise express (N. J. Thrift 2008). Affects “become present only through performance; through the interaction of bodies, spaces, representations and objects” (Spinney 2015, 235). According to these understandings, affects may not be (sufficiently) captured by conventional representational methodologies that build upon people's ability to verbalize or otherwise express their feelings. A key concern that emerged as consequence of NRT and its criticism on conventional representational methods, was the question of how to adequately capture affects and subconscious cognitive processes, something which previous attempts of behavioral geography had failed to do.

Soon after its proposition—which came much as a radical and antipodal perspective to traditional practices of research—there was critique on NRT's deterministic framing of the rational human being as an affect‐driven actor (Barnett 2008). There were also calls to situate and contextualize NRT's findings in relation to people's own voices and subjective meaning making (Hitchings 2012), to acknowledge lasting and more conscious emotions and their representations (Bork‐Hüffer and Yeoh 2017; Merriman 2014) as well as to consider the social, historical, political and academic contexts and discourses within which these findings emerge (Glasze and Mattisek 2021; Pykett 2018). As a results, NRT began to move beyond what Barnett (2008, 189) refers to as “representationalist view on representational practices” and widened its perspective through the lens of more‐than‐representational theories (MRT) and methodologies (Anderson 2019). Shortly thereafter, more and more scholars began not only to advocate for but increasingly to apply innovative research methodologies and designs that encompass both non‐representational and representational methods and thinking (Kaufmann et al. 2021; Laurier and Philo 2006; J. Lorimer 2010). By doing so and by bringing the experiment out of the very laboratory conditions, they so often criticized, some scholars eventually ventured into explorative engagements with diverse biosensors (Osborne and Jones 2017).

### 3.2 Following Mobilities: Mobilities Paradigm

Beyond the challenge of capturing affects, the question of how to research them as they unfold and where they unfold was a related problem. The interest in researching the coming together of bodies, materialities and technologies in situ as well as of everyday (im)mobilities, flows and networks of people and things can also be associated with a mobilities turn and new mobilities paradigm in the social sciences and human geography (Cresswell 2010, 2011; Dirksmeier and Helbrecht 2008; Merriman 2014). Despite a much longer tradition of geographical research into diverse mobilities, particularly in transport geographies, this “turn” was propagated from the mid‐2000s (Cresswell 2010, 2011; Cresswell and Merriman 2010; Merriman 2014). It emerged from “trenchant critiques of the bounded and static categories of nation, ethnicity, community, place, and state within much social science” (Sheller and Urry 2006, 211) which were perpetuated by the ways in which conventional social science methods were researching the social world. As part of this turn, it was claimed that mobility—of people, objects, media, images, knowledge, information, symbols, places, technologies and data—should be recognized and researched as a social norm rather than as exception (Büscher, Urry, and Witchger 2011; Sheller and Urry 2006; Urry 2007). The core methodological concern centers on the question of “how to study mobile phenomena […] without losing sight of what is fascinating about them—mobility” (Kaufmann and Bork‐Hüffer 2021, 316, translated).

Inspired by the anthropological research of Arjun Appadurai, this strand of research was initially more focused on studying the mobilities of material objects in the sense of follow‐the‐thing geographies (Cook 2004; Jackson 2000; Pfaff 2010a, 2010b). Soon after, it began to emphasize engaging with “participants ‘on the move’ in a variety of ways” (Evans and Jones 2011, 849). Overall, methods applied in mobilities research can be broadly defined as “an array of methods that in different ways capture, track, simulate, mimic, parallel and ‘go along with’ the kinds of moving systems and experiences that seem to characterize the contemporary world” (Büscher, Urry, and Witchger 2011, 7). These methods explore mobilities, regardless of whether they are mobile themselves (Kaufmann and Bork‐Hüffer 2021; Moles 2019). In a narrower sense, however, mobile methods can be defined as those that are mobile themselves, following objects, people or data and thus bringing the data collection method to the research context (Büscher and Urry 2009; Hein, Evans, and Jones 2008; Spinney 2009, 2015) with the aim of heightening ecological validity (Birenboim, Helbich, and Kwan 2021). Within human geography particularly qualitative mobile methods, such as walking interviews (Evans and Jones 2011; Jones et al. 2008) have become prevalent.

Less often, quantitative methodologies have been employed, utilizing a range of technologies such as GPS, log data extraction programs, tracking apps and, importantly, biosensors installed on mobile devices like smartphones, smartwatches, wristbands or eye‐trackers (Birenboim, Helbich, and Kwan 2021; Birenboim and Shoval 2016; Giannotti and Pedreschi 2008). Birenboim et al. (2019) used wearable biosensors to measure heart rate, heart rate variability, and skin conductance during an outdoor walk to compare participants' physiological responses when they passed through various types of green, blue and gray urban environments. As part of the so‐called DigitAS (The Digital, Affects and Space) project, a mixed methods study that included eye‐tracking in a mobile quasi‐experimental field study was conducted to analyze the effects of location‐based media content on participants' perceptions of public parks (Kaufmann et al. 2021; Kaufmann and Bork‐Hüffer 2021; Kollert et al. 2021). Inspired by MRT and the mobilities paradigm, the study aimed to combine an analysis of the mobile in situ entanglement of people, technologies and materialities with a comparison of the more‐than‐representational tracking of affective eye movements and people's representations of their emotional experiences of the parks (through retrospective think‐alouds).

There is therefore certainly a compelling argument that the mobility paradigm has provided scholars experimenting with wearables and biosensing a theoretical and epistemological foundation that might have fostered the openness toward a method(ology) that not only allows for the observation of mobilities but is also mobile in itself (Pykett 2018).

### 3.3 Attending to Visualities: From Visual Geographies to Geography as a Ocularcentric Discipline

Building on the previously outlined understanding of eye‐tracking as both a biosensing technology and methodology, one can trace the roots of interest to conceptualizations of geography as a visual and ocularcentric discipline (Driver 2003; Rose 2003). A discipline which, as argued by Rose (2003, 212) “has relied and continues to rely on certain kinds of visualities and visual images to construct its knowledges.” While early discussions on the importance of visualizations in geography (Crang 2003; Rose 2003) and its visual turn (Thornes 2004) focused more on the analysis of images and photography, and related questions of aesthetics, performance and power relations, later work—influenced by more‐than‐representational thinking—highlighted the discipline's affinity of “doing and engaging with imagery” (Tolia‐Kelly 2012, 135) and “the multisensual fluidity and rhythms of everyday life” captured by videography (Garrett 2011, 522). Scholars have outlined the potential of videography as a method and medium that enables both participants and researchers to capture and visualize experiences, encounters and mobilities in entangled spaces (Garrett 2011; Heath, Hindmarsh, and Luff 2010; Pink 2021). Equipping participants with a (e.g., handheld, head‐mounted) camera gives them agency to record wherever they choose to direct the sensor. When speaking of geography as a visual discipline then, it is unsurprising that scholars have started to engage with mobile eye‐trackers, as these not only capture snapshots of places, encounters and experiences, but also visualize where the gaze has been directed. In doing so, they allow researchers to not only “look alongside” (Garrett 2011, 534) but to see through the eyes of the participant. The gaze‐enriched recordings offer insights into participant's embodied experiences of their spatial environment, as well as their human and non‐human interactions, without requiring the researcher to be present in the moment. The potential of measuring eye movements in mobile, dynamic “out of the lab” environments and spaces has already been demonstrated by numerous studies in environmental psychology, GIS science and increasingly in geography (see also Table 1). In addition to the aforementioned DigitAS project, Amati et al. (2018) examined the influence of natural features in urban environments on viewing behaviors while strolling to a public park. Kiefer et al. (2017) discussed some of the prevailing challenges of integrating eye‐tracking in GIS and spatial science in general. Teo and Pua (2022) utilized eye‐tracking to research participation processes in inclusive classrooms. Presenting a spatial imagining of compulsivity, Beljaars (2019), conducted interviews, participant observations as well as mobile eye‐tracking with people diagnosed with Tourette syndrome.

**TABLE 1:** Wearable biosensors and their application fields in geography and related fields of interest.

Technology	Body location	Sensor	Measures/Biosignals	Geographical and related studies
Mobile eye‐tracker	Head	Near infrared light (NIR)	Eye movement	Beljaars (2019), Kaufmann and Bork‐Hüffer (2021), Kiefer et al. (2017), Kollert et al. (2021), Melchert and Misera (2022), and Teo and Pua (2022)
		Video camera	Optical	
		Inertial measurement unit (IMU) including accelerometer, gyroscope, magnetometer	Body movement	
		Microphone	Speech/Voice/Acoustics	
Wearable smart device	Finger‐(tip)	Electrodermal activity (EDA)/Galvanic skin response (GSR)	Skin conductance	Berger and Dörrzapf (2018), Caviedes and Figliozzi (2018), Chrisinger and King (2018), Gravenhorst et al. (2012), Nold (2009), Osborne and Jones (2017), and H. Zhang et al. (2022)
	Ear‐(lobe)			
	Wrist			
	Wrist, Chest	Photoplethysmography (PPG)	Heart rate (HR)	Kim and Fesenmaier (2013), S. Li et al. (2015), Paül I Agustí, Rutllant, and Lasala Fortea (2019), Resch et al. (2014), Winz et al. (2022), and Zeile et al. (2016)
			Blood pressure (PB)	
			Puls rate (PR)	
			Pulse rate variability (PRV)	
			Blood oxygen saturation level (BVP) (SpO2)	
		Thermometer	Skin temperature	
(Mobile) Electroencephalogram (EEG)	Head	Scalp‐placed electrodes	Electrical neuron activity (brain)	Aspinall et al. (2015), W. Lin et al. (2020), Mavros, Austwick, and Smith (2016), McMahan, Parberry, and Parsons (2015), F. Zhang et al. (2018), and J. Zhang et al. (2023)

### 3.4 Capturing the Neural and Bio‐Social Links: From Biopolitics to Critical Neurogeographies and the Biosocial Model

Another theoretical debate is linked to the increased interest in biosensing methodologies: the growing influence of neuroscientific thinking on both, urban policy making and the individual—fostered by the commercialization of neurotheologies such as wearable biosensors –, has led to calls for critical reflection upon the consequences of these influences. This body of research focused on critical perspectives on “[n]europolitanism” (Fitzgerald, Rose, and Singh 2016), “neuro‐urbanisms” (Pykett 2013) or the “biosocial” (Winz and Söderström 2021). Pykett (2018, 164), drawing on more‐than‐representational thinking—including the related emergence of affect theories, with poststructuralist thinking, and related theorizations of the governing of bodies through biopolitics—suggested critical neurogeographies as a field “informed by, but not indebted to, neuroscience and cognitive science.” Like more‐than‐representational perspectives, critical neurogeographers argue that there “is the need for analytical approaches which can switch between paradigms, and productively engage with (rather than attempt to resolve) the deep‐rooted historical conflict between positivist and interpretivist forms of analysis” (Pykett et al. 2020, 4–5). They have started to methodologically experiment with “neurotechnologies and wearable biosensors outside of laboratory contexts” (Pykett 2018, 165). Unlike common (critical) human geographical approaches, critical neurogeographies do not emanate from the individual or body as the starting point of analysis but rather from the brain and human cognition. The brain and cognition are thus analyzed and conceptualized within their embeddedness in the social milieu and discourses.

This line of thinking is also prevalent in discussions on biosociality or visceral geographies, which have been brought forward by scholars such as Hayes‐Conroy (Hayes‐Conroy 2010, 2017), Osborne and Jones (2017), Prior, Manley, and Sabel (2019) or Winz and Söderström (2021). Central to their argument is the call “to develop more equally‐balanced bio/social approaches” (Winz and Söderström 2021, 170) and mixed‐method methodologies that acknowledge the complexities and entanglements of biological processes and social pathways—something which can be achieved by integrating more‐than‐representational biosensing or embodied “visceral methods” (Hayes‐Conroy 2017) in representational research designs (Osborne and Jones 2017).

### 3.5 Experimenting With Biosensors

It should be noted that many geographic and spatial ventures into biosensing have been driven less by theoretical debates, such as those introduced above, and more by an interest in methodological experimentation and innovation. These endeavors have utilized a broad range of biosensors. While there have been efforts to summarize existing geography‐related studies engaging with biosensing (Osborne et al. 2023; Osborne and Jones 2017; Paiva et al. 2023) these studies have not been categorized based on the specific technology and sensors used. This is evident in Table, which includes many of the studies mentioned in previous sections.

In general, the increase of scholarly interest in experimenting with biosensors can be attributed to advancements in technology, as well as the increasing accessibility and affordability of wearable biosensors in the past decade. With the exception of early pioneers such as Nold's (2009) innovative approach of integrating GSR and GPS measures on maps, Gravenhorst et al.'s (2012) work on monitoring electrodermal activity in daily life, or Ward Thompson et al.'s (2012) stress‐related examination of salivary cortisol patterns in green spaces, most studies have emerged since the mid‐2010s. This surge has been driven by a growing interest in researching affective and emotional responses within different environments as well as a desire to explore innovative mobile and more‐than‐representational methods (Pykett 2018; Spinney 2015). Most of this early work has focused on urban emotion, mental health as well as stress. It has experimented with wrist‐ or ankle‐worn wearables that allow the simultaneous measurement of various somatic responses such as galvanic skin response (GSR) and heart rate (HR)—likely due to their relative affordability (Kim and Fesenmaier 2013; S. Li et al. 2015; Resch et al. 2014; Zeile et al. 2016). Some scholars have examined cognitive responses in virtual environments using head‐mounted EEGs (McMahan, Parberry, and Parsons 2015; B. Zhang 2018), while others have utilized them in outdoor environments (Aspinall et al. 2015; W. Lin et al. 2020; Mavros, Austwick, and Smith 2016). EDA/GSR sensors have been applied in study conditions, where participants were guided to follow a certain route or trajectory (Berger and Dörrzapf 2018; Caviedes and Figliozzi 2018; Chrisinger and King 2018).

## 4 Applications: Potentials and Limits

While biosensing offers promising avenues for geographers, it also presents challenges that scholars need to carefully reflect upon, especially as both its application and development are currently “outpacing the science required to rigorously interpret the data generated” (Pykett et al. 2020, 1). These challenges involve practical issues stemming from the complexity of each sensor and restrictions on their use, and the specific data they produce. It is therefore hardly possible to provide a comprehensive overview of all potential challenges. Nonetheless, we provide a brief overview of some of the most critical potentials and challenges associated with specific wearable biosensors.

Although frequently used by geographers, EDA and GSR sensors, along with thermometers, present three crucial constrains. Firstly, the biosignals measured by these devices do not provide any recording and evidence of the specific environmental stimuli that caused a reaction. Secondly, any biochemical response to a stimulus always occurs with a certain delay (Kyriakou et al. 2019). Thirdly, these sensors are highly susceptible to noisy signals, as our skin produces moisture even after the slightest movements and activities (Blasco and Peris‐Lopez 2018). It must thus be carefully considered whether these devices can provide valid results, especially when used outside a lab environment. In contrast to biosignals measured by EDA sensors, eye movements occur almost instantaneously in response to a stimulus (Skaramagkas et al. 2021). They are measured by optical sensors in mobile eye‐trackers, which have integrated video cameras that allow researchers to track participant's gazes and determine whether the participants have noticed certain stimuli (Pérez‐Edgar, MacNeill, and Fu 2020). Although current generation devices have resolved many of the previous limitations, such as a low Hz rates, and are increasingly suited for outdoor environments, they still tend to rely on proprietary algorithms developed by software companies (Valtakari et al. 2021). In comparison to the lightweight design of mobile eye‐trackers which are hardly distinguishable from regular glasses, the design of mobile EEGs raises questions whether they can truly provide ecological validity when used in outdoor environments (Birenboim et al. 2019).

In general, care must be taken when attributing spikes or patterns of somatic responses to stimuli in “messy, naturalistic environments” (Harvey 2024, 1), particularly since there remains a limited understanding of the relationship between biosignals and emotions (Poplin 2020). This has also been highlighted by Brigstocke et al. (2023, 590) who argue that the quantitative information gathered by biosensors and influenced by uncontrollable variables during mobile use, does not provide information “about the quality of the affect, or how it is experienced at a subjective level.” While further technological improvements in wearable biosensors may address other current constraints such as battery life, contamination or the inferior quality of many biochemical sensors and procedures, the challenge of “making sense” of the vast datasets generated by these devices remains.

It is therefore advisable to combine biosensor measurements with qualitative methodologies as part of mixed methods approaches (Osborne and Jones 2017). For geographers, the key contribution here—one that would significantly complement existing studies in other disciplines—is to contextualize biosensing data. A more‐than‐representational analysis of bodies, their (somatic) responses, and affects needs to be situated within analyses of emotions, power relations, asymmetries and related inequalities and (im‐)mobilities. Qualitative methods such as (retrospective) think‐alouds, go alongs, participant narratives and pre‐ or follow‐up interviews allow the comparison of measured affects with participants' own accounts and verbalized emotions (Birenboim et al. 2019; Hall and Bates 2019; Kaufmann and Bork‐Hüffer 2021). Additional analysis of socio‐political discourses, stakeholder and expert interviews or focus groups can provide insights into the socio‐political space in which the research is situated. Observations and video content analysis (e.g., the case of eye‐tracking) can help uncover material dimensions. In this way, mixed methods approaches can address some of the critique directed toward early studies and their respective behavioral models.

## 5 Toward an Ethics of Care in Biosensing

Our overview revealed that geographers have so far focused on a narrow range of biosensing technologies and few biosensors. Most have measured EDA, GSR or PPG signals using wearable smart devices. Although there has been a number of studies using EEGs and mobile eye‐trackers in spatial research, few have had geographers among their authors. As other scholars and we have argued, the use of wearable biosensors within mixed methods approaches presents an opportunity to explore the coming together of bodies, material and technological relations, their contextualities and performativities and the associated affects, emotions and (im‐)mobilities while they unfold (Büscher and Urry 2009; Spinney 2015; Kaufmann and Bork‐Hüffer 2021). This should be of significant interest to scholars working with relational, more‐than‐representational, (new) materialist, posthumanist, mobilities, biosocial and visual geography perspectives, across a broad spectrum of research themes and questions.

Given the complexity of biosensing, many geographers have collaborated with scholars from other disciplines mutually benefitting from each other's expertise. However, just as diverse as these interdisciplinary engagements but also the studies, fields of application and publications in other disciplines (see Section 2) are, so are the terminologies and understandings. Gaining an overview of the variety of biosensors and their varying potentials and challenges is time‐consuming and demanding. Methodologies and applications are informed by a variety of theoretical paradigms.

Importantly, this diversity brings challenges not only in terms of differing terminologies and methodologies, but also with respect to ethical standards (Balsamo and Mitcham 2010; Geballa‐Koukoula et al. 2023). Thus, in addition to geographers' important efforts toward the spatial contextualization of biosensing research (see Section 4), we call for attention to the mobilization, development, implementation and debate of research ethics in biosensing. While institutional ethics procedures and data management practices have been continuously refined over the last 2 decades, we postulate that current supranational (e.g., EC 2021; ALLEA – All European Academies 2023) and institutional guidelines, with their focus on social science research and data management, are too superficial to provide adequate guidance for the use of biosensors in mobile, out‐of‐laboratory settings, and for managing the data these sensors produce. Hence, we argue that applying biosensors in the field requires not only strict adherence to institutional ethics procedures (among others proper informed consent), but also the incorporation of data ethics, procedural ethics, and an ethics of care.

Many biosensing technologies integrate several biosensors within a single device, collecting diverse streams of data simultaneously, much of which is often left unanalyzed. With regard to data ethics and management this is, of course, an issue. If it is not possible to deactivate some of the unwanted measures before the start of the recording, researchers should ensure that any irrelevant data is deleted immediately afterward. Researchers should also be aware that the data collected by EEG sensors is personally‐identifiable. Even if data from other sensors may not be personally identifiable on its own, the collection of carious biosignals may enable the tracing and identification of individuals, especially when presented together (Yang et al. 2022). A careful approach to data management becomes particularly urgent when experimenting with mobile eye‐trackers as they enable both video and audio recording. Participants' voices and, occasionally parts of their bodies (such as feet, legs and hands) are recorded, along with bystanders captured by the video camera or audio recorder. Here, a highly sensitive approach to visual and audio data is necessary. Furthermore, it is important to consider that most data collected through biosensors relates to states of health, well‐being and disease (Andreoletti et al. 2024). As geographers lack medical expertise and are not able to detect health‐problems, they should provide participants with contacts for professional medical care in the informed consent. Hence, both data security and privacy concerns must be carefully addressed when handling biosensing data. Data management should comply with FAIR data procedures (cf. Wilkinson 2016) and (non‐) accessibility should be carefully considered and clearly outlined in the informed consent.

Data ethics also encompasses the use of appropriate and secure analysis software. However, such software is often still under development and becoming increasingly complex. In order to deal with large and diverse data sets, more and more companies have started to integrate machine learning, deep neural networks, natural language processing and predictive analytics into their software solutions (Andreoletti et al. 2024). Avoiding gender, racial, age and other biases in advanced data analytics is a serious challenge that is not yet sufficiently addressed in this field to our knowledge. Preventing misuse of data is crucial, as biosensing itself can contribute to the very biopolitics it seeks to critique (see Section 3.4). Holding population level datasets concerning affective states could enable the categorization, management and governing of particular populations and matters of concern.

In view of the serious lack of institutional ethics procedures, “doing ethics” or “procedural ethics” (Guillemin and Gillam 2004) become ever more important, particularly when using biosensors as part of complex mixed methods approaches in the field. Procedural ethics can be supported, for example, through social reflection, validation, support, discussion and decisions within the research team and/or through the inclusion of an ethics advisor or an (ethics) advisory board (Kaufmann et al. 2021). Given the complexity of field settings, researchers may need to adopt what Spiel et al. (2020) refer to as “micro‐ethics” spontaneous and flexible adaptations that align research with the specificities of the social, material and technological environment, including stakeholders, power relations, as well as material conditions (such as weather and barriers in the field).

When implementing an “ethics of care” (Burton and Dunn 2013; Maio 2018) in biosensing research, scholars need to attend to participants' experiences and needs throughout the research process. This includes building trust, recognizing emotional knowledge, and acknowledging power relations involved in the researcher‐participant and technology‐participant relationships. Not all participants, of course, may accept biosensors as appropriate research tools. This challenge surfaced, for example, in a study by Nebeker et al. (2017). There, the wrist‐worn technology was perceived by migrant women from different origins in Southern California as either untrustworthy, a legal risk, or at times even incompatible with social norms and religious practices. Therefore, scholars should carefully consider whether biosensing might cause harm or lead to the exclusion of certain population groups. Even when appropriate, the implementation of biosensing demands continuous reflection and awareness of emotional, social, and material boundaries in the field. Neglecting these factors could place participants in uncomfortable, stressfull, and potentially even harmful situations. Additionally, qualitative methods such as interviews or (retrospective) think‐alouds should be used to allow participants the possibility to voice their own experience, feelings and interpretations. Given the relative novelty of these methodologies, participants should be invited to evaluate the methodology and research process to inform future improvements.

Overall, we observe that the development and application of biosensing is vibrant, highly diverse and very promising for a great range of geographical research questions and fields. As the development of biosensing methodologies is still in its early stages, they continue to evolve and expand through new or improved technologies. As our discussion of the potentials and limits of biosensors has shown, we particularly encourage research using eye‐trackers, which come closest to measuring instantaneous responses to environmental stimuli and offer valuable opportunities for the analysis of social, material and technological environments through the visual data they generate. Fully realizing this potential will require meticulous preparation, planning, and the consideration of processual and careful research and data ethics.

## Conflicts of Interest

The authors declare no conflicts of interest.
